# Isolation, identification, and biochemical characterization of a novel bifunctional phosphomannomutase/phosphoglucomutase from the metagenome of the brown alga *Laminaria digitata*

**DOI:** 10.3389/fmicb.2022.1000634

**Published:** 2022-09-23

**Authors:** Stephen A. Jackson, Maohang Duan, Pengyan Zhang, Maureen W. Ihua, Dagmar B. Stengel, Delin Duan, Alan D. W. Dobson

**Affiliations:** ^1^School of Microbiology, University College Cork, Cork, Ireland; ^2^Environmental Research Institute, University College Cork, Cork, Ireland; ^3^Key Laboratory of Sustainable Development of Marine Fisheries, Ministry of Agriculture and Rural Affairs, Yellow Sea Fisheries Research Institute, Chinese Academy of Fishery Sciences, Qingdao, China; ^4^Botany and Plant Science, School of Natural Sciences, Ryan Institute for Environmental, Marine and Energy Research, University of Galway, Galway, Ireland; ^5^CAS and Shandong Province Key Laboratory of Experimental Marine Biology, Center for Ocean Mega-Science, Institute of Oceanology, Chinese Academy of Sciences, Qingdao, China; ^6^Laboratory for Marine Biology and Biotechnology, Pilot National Laboratory for Marine Science and Technology, Qingdao, China

**Keywords:** Macroalga, Phosphoglucomutase, Phosphomannomutase, Metagenomics, *Laminaria digitata*

## Abstract

Macroalgae host diverse epiphytic bacterial communities with potential symbiotic roles including important roles influencing morphogenesis and growth of the host, nutrient exchange, and protection of the host from pathogens. Macroalgal cell wall structures, exudates, and intra-cellular environments possess numerous complex and valuable carbohydrates such as cellulose, hemi-cellulose, mannans, alginates, fucoidans, and laminarin. Bacterial colonizers of macroalgae are important carbon cyclers, acquiring nutrition from living macroalgae and also from decaying macroalgae. Seaweed epiphytic communities are a rich source of diverse carbohydrate-active enzymes which may have useful applications in industrial bioprocessing. With this in mind, we constructed a large insert fosmid clone library from the metagenome of *Laminaria digitata* (Ochrophyta) in which decay was induced. Subsequent sequencing of a fosmid clone insert revealed the presence of a gene encoding a bifunctional phosphomannomutase/phosphoglucomutase (PMM/PGM) enzyme 10L6AlgC, closely related to a protein from the halophilic marine bacterium, *Cobetia* sp. 10L6AlgC was subsequently heterologously expressed in *Escherichia coli* and biochemically characterized. The enzyme was found to possess both PMM and PGM activity, which had temperature and pH optima of 45°C and 8.0, respectively; for both activities. The PMM activity had a *K*_m_ of 2.229 mM and *V*_max_ of 29.35 mM min^−1^ mg^−1^, while the PGM activity had a *K*_m_ of 0.5314 mM and a *V*_max_ of 644.7 mM min^−1^ mg^−1^. Overall characterization of the enzyme including the above parameters as well as the influence of various divalent cations on these activities revealed that 10L6AlgC has a unique biochemical profile when compared to previously characterized PMM/PGM bifunctional enzymes. Thus 10L6AlgC may find utility in enzyme-based production of biochemicals with different potential industrial applications, in which other bacterial PMM/PGMs have previously been used such as in the production of low-calorie sweeteners in the food industry.

## Introduction

*Laminaria digitata* (Hudson) J.V. Lamouroux (Lamouroux, 1813) is a dark-brown seaweed (Laminariales, Phaeophyceae) which is typically distributed in European coastal waters in areas ranging from the southern coast of Brittany in France to Iceland and the north coasts of Scandinavia (Supplementary Figure S1). It is a commercially important species as a source of laminarin, fucoidans, alginates, and nutraceuticals such as glutamic acid, iodine, antioxidants, and vanadium. Laminarin is particularly important being a water-soluble storage polysaccharide with reported anticoagulant (Miao et al., 1995), anti-apoptotic (Kim et al., 2006), antioxidant (Choi et al., 2012), anti-inflammatory (Neyrinck et al., 2007) and anti-tumor activities (Ji et al., 2013; Park et al., 2013).

Marine macroalgae such as *L. digitata* play host to a broad range of epiphytic bacterial communities, with organic substances which are secreted by the macroalga acting as an important nutritional source for these microorganisms (Egan et al., 2013). These epiphytes are known to be involved with not only regulating the macroalgal morphogenesis and growth (Egan et al., 2013), but also in the production of chemical substances which help the macroalgae to protect themselves from potentially harmful secondary colonization involving pathogenic microorganisms (Singh and Reddy, 2014). We previously reported on epibacterial communities from *L. digitata* where different regions of the seaweed hosted distinctly different associated microbial populations (Ihua et al., 2020).

Marine environments are a particularly interesting source of novel biocatalysts primarily due to the varied and quite extreme conditions to which the marine biome is exposed, such as high salinity, high-pressure levels coupled with variations in pH and low temperature; which has resulted in extremophilic microorganisms that have adapted to these severe conditions and which possess enzymes that function under atypical reaction conditions (Castilla et al., 2018).

Marine bacteria which degrade algal polysaccharides play an important role in both the global carbon cycle and in algal biomass recycling in particular (Azam et al., 1998). Previous reports have highlighted that bacteria associated with the macroalga *Ascophyllum nodosum* are enriched in macroalgal-polysaccharide-degrading bacteria, which possess agarase, κ-carrageenase, ι-carrageenase, and alginate lyase activities (Martin et al., 2015). Subsequent functional screening of plurogenomic libraries from these bacteria resulted in the identification of a broad range of novel hydrolytic enzymes (Martin et al., 2016). Our group has also previously reported on the isolation of bacteria from decaying samples of *A. nodosum* which displayed multiple hydrolytic enzyme activities, that were subsequently used in the enzyme-assisted extraction (EAE) of phenolics from the macroalga *Fucus vesiculosus* (Ihua et al., 2019). Therefore microbiota associated with macroalgae such as *A. nodosum* clearly constitute an interesting source of novel carbohydrate-active enzymes and could potentially be applied for enzyme-assisted extraction (EAE) strategies and improvements in the yields of algal components with functional food, nutraceutical, biopharmaceutical and cosmetics applications (Ihua et al., 2019).

The microbiome of these macroalgae contain an array of bacteria with potentially interesting carbohydrate-active enzymes, however given that typically only 1% of marine bacteria are cultivable by employing standard laboratory protocols (Glöckner and Joint, 2010), it is highly likely that the macroalgal microbiome is a good source of as yet undiscovered novel carbohydrate-active enzymes.

Metagenomic approaches are typically employed to exploit the unculturable fraction of different environmental ecosystems, (Vieites et al., 2009), where metagenomic DNA (mDNA) is extracted from the environmental sample and analyzed using high throughput sequencing technologies to identify potential targeted enzymes (Dinsdale et al., 2008), which can then be cloned into heterologous expression systems and screened for the desired phenotype (Kennedy et al., 2011; Coughlan et al., 2015; Garcia-Moyano et al., 2021; Newgas et al., 2021). Alternatively, mDNA can be cloned directly into a suitable vector in heterologous hosts such as *Escherichia coli*, *Streptomyces lividans*, *Pseudomonas putida,* and *Bacillus subtilis* (Lam et al., 2015; Zhang et al., 2017), thereby generating a metagenomic library which can then be screened using phenotypic based assay systems to detect the newly acquired phenotype conferred on the host by the metagenomic clone (Coughlan et al., 2015; Lam et al., 2015; Ngara and Zhang, 2018). Such approaches have been used to date to identify novel biocatalysts from the microbiomes of various different marine ecosystems ranging from marine mud and sediment (Lee et al., 2015; Gao et al., 2016), deep sea sediment (Huo et al., 2018), shrimp (Park et al., 2011), marine sponges (Selvin et al., 2012; de Olivera et al., 2020) to the aforementioned macroalga *A. nodosum* (Martin et al., 2014). Functional metagenomic approaches from marine environments have to date resulted in the isolation of novel lipases, esterases, phosphatases, and cellulases among others, with potential industrial applications (Ngara and Zhang, 2018; de Oliveira et al., 2021).

With this in mind, we targeted the microbiome of *L. digitata* to identify novel carbohydrate-active enzymes. mDNA was isolated from *L. digitata* which was allowed to decay at three different temperatures, namely 18, 25, and 30°C, over a 6-week period, and a metagenomic large-insert fosmid clone library was subsequently constructed. Fosmid insert sequencing from one metagenomic clone revealed the presence of an interesting bifunctional phosphomannomutase/phosphoglucomutase (PMM/PGM) enzyme which we subsequently sub-cloned, expressed, and biochemically characterized. PMM/PGM are members of the alpha-d-phosphohexomutase superfamily of enzymes and catalyze the reversible conversion of 1-phospho to 6-phosphohexoses, involving various different sugars such as glucosamine, glucose, mannose, and N-acetylglucosamine. Optimum enzyme activities for both PGM and PMM activities were observed at 45°C and pH 8.0. The highest PGM activity was observed with Mg^2+^ as a co-factor but PMM activity was highest with Mn^2+^.

## Materials and methods

α-d-Glucose-1-phosphate disodium salt (G1P), α-d-Mannose-1-phosphate sodium salt (M1P), and α-d-Glucose-1, 6-diphosphate potassium salt were purchased from Biosynth Carbosynth (United Kingdom). NADP and NADPH were purchased from Sangon Biotech (China). Phosphoglucose isomerase, phosphomannose isomerase, and glucose-6-phosphate dehydrogenase were purchased from Sigma-Aldrich (United States).

### Sampling and induced decay

*Laminaria digitata*, including blade, meristem, stipe, and holdfast regions, was collected from Finavarra in Co. Clare, Ireland, at 53° 08′ 59″ N, 9° 08′ 09″ W, as previously described (Ihua et al., 2020). All samples were kept on dry ice at the sampling location and subsequently stored at either −20°C or −80°C. No permits were required for sample collection as the sampling location is open to public access and use, and the alga was harvested in small quantities. Artificially induced decay of the seaweed was achieved by adding approximately 450 g of *L. digitata*, roughly chopped (2 cm × 10 cm), to 950 ml sterile artificial seawater (3.33% *w/v* Instant Ocean, Aquarium Systems, FL, United States), in each of three 2 l flasks and the flasks were incubated at different temperatures (18, 25, and 30°C) on a shaking platform at 125 rpm for a 6-week period. Triplicate 5 g samples were withdrawn from each flask after 2, 4, and 6 weeks. Samples were stored at −80°C until further use.

### DNA extraction

Total mDNA was extracted individually from 1 to 1.5 g of each of 30 samples (triplicate samples from 0 weeks decay and triplicate samples from each incubation temperature after 2, 4, and 6 weeks decay). Samples were thawed on ice and suspended in 1 ml of lysis buffer (100 mM Tris, 0.5 M EDTA, 100 mM NaH_2_PO_4_, 5 M NaCl, and 10% CTAB) and incubated for 2 h at 70°C. Each sample was split in half and 1 ml of chloroform: isoamyl-alcohol (24:1) was added to each in 2 ml Eppendorf tubes, mixed by vortexing, and then centrifuged at 9,000 × *g*. The upper phase was transferred to fresh Eppendorf tubes and 0.7 vols isopropanol and 0.1 vols 3 M sodium acetate pH 5.2 was added to each tube. Tubes were inverted several times to mix and were then centrifuged for 30 min at 25,000 × *g*. Supernatants were discarded and pellets were washed with cold (4°C) 70% EtOH (500 μl EtOH was added to each tube and was centrifuged for 10 min at 25,000 × *g*). Ethanol was discarded and the pellets were air-dried at room temperature and then dissolved in TE buffer. Each DNA sample was treated with proteinase K and RNase A. All samples were then pooled. An equal volume of phenol: chloroform: isoamyl alcohol (25:24:1) was added. Tubes were centrifuged at 25,000 × *g* for 30 min. The upper phase was transferred to fresh tubes and the DNA was precipitated with isopropanol and sodium acetate as before and washed with cold ethanol as before. DNA was quantified by NanoDrop ND-1000 and viewed by electrophoresis on a 1% agarose gel.

### DNA fractionation

The HMW DNA was purified and size-separated by pulsed-field gel electrophoresis (PFGE) at 6 V/cm, 1–25 s switch time, 120° angle, 17 h, 14°C in 0.5X TBE buffer (45 mM Tris–HCl, 45 mM boric acid, 1 mM EDTA, pH 8.3) on a 1% PFGE grade agarose gel (Bio-Rad Laboratories). For size comparison, each gel contained a PFGE DNA Molecular marker (MidRange II PFG Marker, New England Biolabs). The marker lane and approx. 0.5 cm of the sample DNA lane were cut off and stained for 30 min in SafeView dye for visualization. A gel slice containing unstained DNA of about 30–50 Kb was excised and the DNA was electro-eluted from the gel slice and concentrated as described by Brady (2007), with the following modifications: Electro-elution was performed for 3 h with replacement of running buffer after 90 min; DNA was concentrated to a final volume of approximately 100 μl with VivaSpin 6 (MWCO 50,000) concentrator tubes (Sartorius). Washed and concentrated DNA was dislodged from the membranes by placing the filter upside-down in a clean 50 ml tube and centrifuging for 5 min at 5,000 × *g*. Size and purity were checked by PFGE using the conditions stated above. DNA concentration was determined using a NanoDrop ND-1000 spectrophotometer.

### Construction of metagenomic fosmid library

Fractionated HMW DNA was end-repaired using the End-It™ end repair kit (Epicentre) according to the manufacturer’s instructions and metagenomic libraries were constructed using the vector pCCERI, a fosmid derived from the commercial pCC1FOS (Epicentre Biotechnologies) vector (Selvin et al., 2012). The circular vector was linearized using *BstZ*17*I* (New England Biolabs, United Kingdom) and dephosphorylated using shrimp alkaline phosphatase (New England Biolabs, United Kingdom) and subsequently purified by phenol extraction after inactivating the enzymes by incubation at 70°C. A ligation reaction was performed with ~0.25 μg insert (20–50 Kb mDNA) and ~0.5 μg linearized vector T4 ligase (New England Biolabs, United Kingdom). Ligation reaction products were packaged in Lambda Phage packaging extracts (MaxPlax™, Lucigen) and transfected into *E. coli* EPI300™ host cells. The transfection reaction products were spread to Q-Trays on LB agar including kanamycin (50 μg/ml) and chloramphenicol (12.5 μg/ml) and clones were grown by incubating at 37°C. Clones were hand-picked from isolated colonies into LB broth (including kanamycin and chloramphenicol) in 384 well microplates (Genetix) and grown by incubating at 37°C. Subsequently clones were replicated to 384 well microplates containing storage medium [0.1% tryptone (*w/v*), 0.05% yeast extract (*w/v*), 0.05% NaCl (*w/v*), 0.63% K_2_HPO_4_ (*w/v*), 0.018% KH_2_PO_4_ (*w/v*), 0.005% sodium citrate dehydrate (*w/v*), 0.009% (NH₄)₂SO₄ (*w/v*), 0.0009% MgSO_4_.7H_2_O (*w/v*), and 0.6% glycerol (*v/v*)] for long-term storage.

### Fosmid extraction and sequencing

Clone 10L6 was grown overnight in LB broth supplemented with kanamycin (50 μg/ml), chloramphenicol (12.5 μg/ml), and 0.1% arabinose. Cells were pelleted and the fosmid was extracted with the FosmidMax (Lucigen) extraction kit, according to the manufacturer’s instructions. Fosmid (including contaminating genomic DNA) was sequenced by Illumina HiSeq (Eurofins Genomics Ltd., Germany). Sequences were assembled by Eurofins Genomics Ltd., using *de novo* assembly with CLC Genomics Workbench (version 4.22.107090). Assembled contigs were annotated using PROKKA v.3.2.1 (Seemann, 2014) on the KBase web platform (Arkin et al., 2018).

### Expression of 10L6AlgC

The 10L6*algC* gene sequence (Accession No.: ON876002) was codon optimized for expression in *E. coli* and synthesized by Sangon Biotech (China). The *algC* was sub-cloned into the *NdeI-EcoRI* sites of the pMAL-c5X vector (New England Biolabs, United States) to allow for the production of PMM/PGM containing a maltose-binding protein (MBP) tag at the N-terminus. The recombinant plasmid was subsequently transformed into competent *E. coli* BL21 (DE3) (Vazyme Biotech, China). Cultures were grown at 37°C until OD_600_ reached 0.6, and induced with 0.3 mM isopropyl-β-d-thiogalactopyranoside (IPTG) for 16 h at 16°C. The cells were harvested by centrifugation at 8,000 rpm for 10 min at 4°C, and pellets were rapidly frozen with liquid nitrogen and stored at −80°C.

### Purification of 10L6AlgC

Purification was performed on the MBPTrap HP 5 ml column (Cytiva, United States) using ÄKTA Pure (GE Healthcare, United States). Cell paste which was obtained following centrifugation of 500 ml of culture was resuspended in 30 ml binding buffer (20 mM Tris–HCl, 200 mM NaCl, and pH 7.4) and was sonicated until the solution was clear. The supernatant was obtained by centrifugation at 13,000 rpm for 40 min and purified using the MBPTrap HP column which had been equilibrated with binding buffer (pH 7.4). PMM/PGM was eluted from the column by washing with binding buffer supplemented with 20 mM maltose. The eluted protein was further purified with a HiLoad™ 16/60 Superdex™ 200 pg. column (GE Healthcare, United States), which had been pre-equilibrated with buffer containing 20 mM Tris–HCl (pH 7.4), and 150 mM NaCl. Fractions containing PMM/PGM were identified by SDS-PAGE. The concentrations of the protein fractions were measured by UV–Vis Spectrophotometer (DeNovix, United States).

### Enzyme activity assays

The PMM and PGM activities of 10L6AlgC were measured using the method described by Zhang et al. (2018). The absorbance of NADPH at 340 nm ultraviolet light was monitored by the coupling assay with glucose-6-phosphate dehydrogenase using PowerWave HT microplate spectrophotometer (BioTek, United States). Standard reaction solution for PGM assays contained 50 mM Tris–HCl, 10 mM MgCl_2_, 1 mM NADP^+^, 0.1 mM glucose-1,6-bisphosphate, 1 mM G1P, and 0.5 U glucose-6-phosphate dehydrogenase. PMM activity was measured in a similar mixture by replacing G1P with 1 mM M1P and supplementation with 0.5 U phosphoglucose isomerase and 0.5 U phosphomannose isomerase. A NADPH standard curve for both PGM and PMM activity was generated with a series of standard solutions at concentrations of 0, 0.1, 0.2, 0.3, 0.4, 0.5, and 1.0 mM, respectively (Supplementary Figure S1).

The optimum temperature for activity was determined by monitoring enzyme activity at 25, 30, 35, 40, 45, 50, and 55°C in 50 mM Tris–HCl (pH 7.5), respectively. The optimum pH value was determined by measuring the reaction in mixtures at pH values of 6.5, 7.0, 7.5, 8.0, 8.5, 9.0, and 9.5 at 45°C. To study the effects of metal ions, MgCl_2_ was replaced by 5 mM NaCl, LiCl, CoCl_2_, MnCl_2_, CaCl_2_, NiCl_2_, and ZnCl_2_ in the reaction mixtures. The thermal stability of the protein was measured by incubating 10L6AlgC at different temperatures (45, 50, 55, 60, 65, and 70°C) for 10 min and then assaying the PMM/PGM activities in the standard reaction. The effect of NaCl concentration on PMM/PGM activity was measured in mixtures of 0, 0.1, 0.2, 0.3, 0.4, 0.5, 0.6, and 0.7 M NaCl, respectively. All the aforementioned experiments were conducted in triplicate.

Kinetic analysis was conducted by adding G1P ranging from 0 to 2 mM and M1P ranged from 0 to 4 mM M1P, respectively, at optimal reaction condition, with each reaction being conducted in quadruplicate. Kinetic parameters were fitted to the Equation $V=\frac{V_{\max}\left[S\right]}{K_{m}+\left[S\right]\left(1+\left[S\right]/K_{i}\right)}$ (Naught and Tipton, 2001) and calculated using GraphPad Prism 8,^1^ based on nonlinear regression of the least square method.

### Protein structure prediction

The SWISS-MODEL template library (SMTL version2022-06-29, PDB release 2022-06-24) was searched with BLAST (Camacho et al., 2009) and HHBlits (Steinegger et al., 2019) for evolutionary-related structures matching the target sequence. The target sequence was searched with BLAST against the primary amino acid sequence contained in the SMTL. A total of 46 templates were found. An initial HHblits profile was built using the procedure outlined in Steinegger et al. (2019), followed by 1 iteration of HHblits againstUniclust30 (Mirdita et al., 2016). The obtained profile was then searched against all profiles of the SMTL. A total of 300 templates were found. The ligands in the template structure was transferred by homology to the model, as it is annotated as biologically relevant, is in contact with the model, and is not clashing with the protein, and the residues in contact with the ligands are conserved between the model and the template. The global and per-residue model quality was assessed using the QMEAN scoring function (Studer et al., 2020).

### Sequence analysis

Protein sequence alignments were performed with (i) T-Coffee (Notredame et al., 2000) and (ii) MUSCLE (Edgar, 2004). Structural alignments were performed with ESPript 3.0 (Robert and Gouet, 2014). Phylogenetic tree building was performed with MEGA X (Kumar et al., 2018). Subcellular localization was inferred with PSORTb version 3.0.3 (Yu et al., 2010).

## Results

### Clone 10L6 sequence analysis

Illumina HiSeq sequencing yielded 4,235,950 reads which were assembled to 2,932 contigs, with a coverage of 290x over 4,366,821 sites. These included 67 contigs larger than 10 Kb, 76 contigs between 5 and 10 Kb in length, and 2,789 contigs smaller than 5 Kb. BLAST analyses of all contigs revealed sequences which mapped to (i) a helper plasmid sequence with 99.95% sequence identity to pRH220 (accession no.: AB526840) from the cloning host, (ii) a complete cloning vector (pCCERI) sequence (12.3 Kb) (Supplementary Figure S2), (iii) contigs ranging in size from 218 b to 12.9 Kb which all mapped to *E. coli* chromosomal sequences, and (iv) a cloned fosmid insert sequence of 34,905 bp in length which shared 99% similarity (94% query coverage) with a *Cobetia* sp. (strain L2A1) complete genome sequence (accession no. CP047025).

**Figure 1:**
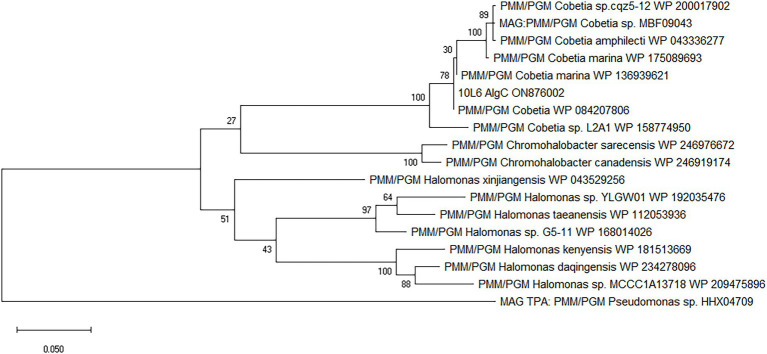
Amino acid-based maximum likelihood phylogenetic tree of 10L6AlgC and related proteins. The evolutionary history was inferred by using the maximum likelihood method and JTT matrix-based model (Jones et al., 1992). Bootstrap consensus (Felsenstein, 1985) was performed by 100 iterations and the bootstrap values are displayed at the nodes of the tree. The tree is drawn to scale, with branch lengths measured in the number of substitutions per site. Evolutionary analyses were conducted in MEGA X (Kumar et al., 2018).

The cloned insert was analyzed using the BASys web portal (Van Domselaar et al., 2005)^2^ and 26 COGs were identified (Supplementary Figure S3; Supplementary Table S1). Genes were identified with potential roles in translation, transcription, transport, signaling, cell division, and lipid metabolism as well as genes of unknown function and a phosphohexose mutase gene (*algC*), potentially involved in carbohydrate metabolism (Supplementary Table S1). Subsequent BLAST analysis showed that this gene was a potential member of the PMM/PGM family enzyme (EC 5.4.2.2/EC 5.4.2.8). BLAST analysis of the nucleotide sequence revealed the closest known related gene sequence to be from the aforementioned *Cobetia* sp. L2A1 (99.5% similarity). The translated protein consisted of 460 amino acid residues. The protein was similar to other PMM/PGM proteins from *Cobetia* spp., including *Cobetia marina*, and also displayed similarities to proteins from *Chromohalobacter* spp. and *Halomonas* spp., and to *Chromohalobacter* spp. enzymes (Figure 1).

### Expression and purification of 10L6AlgC

**Figure 2:**
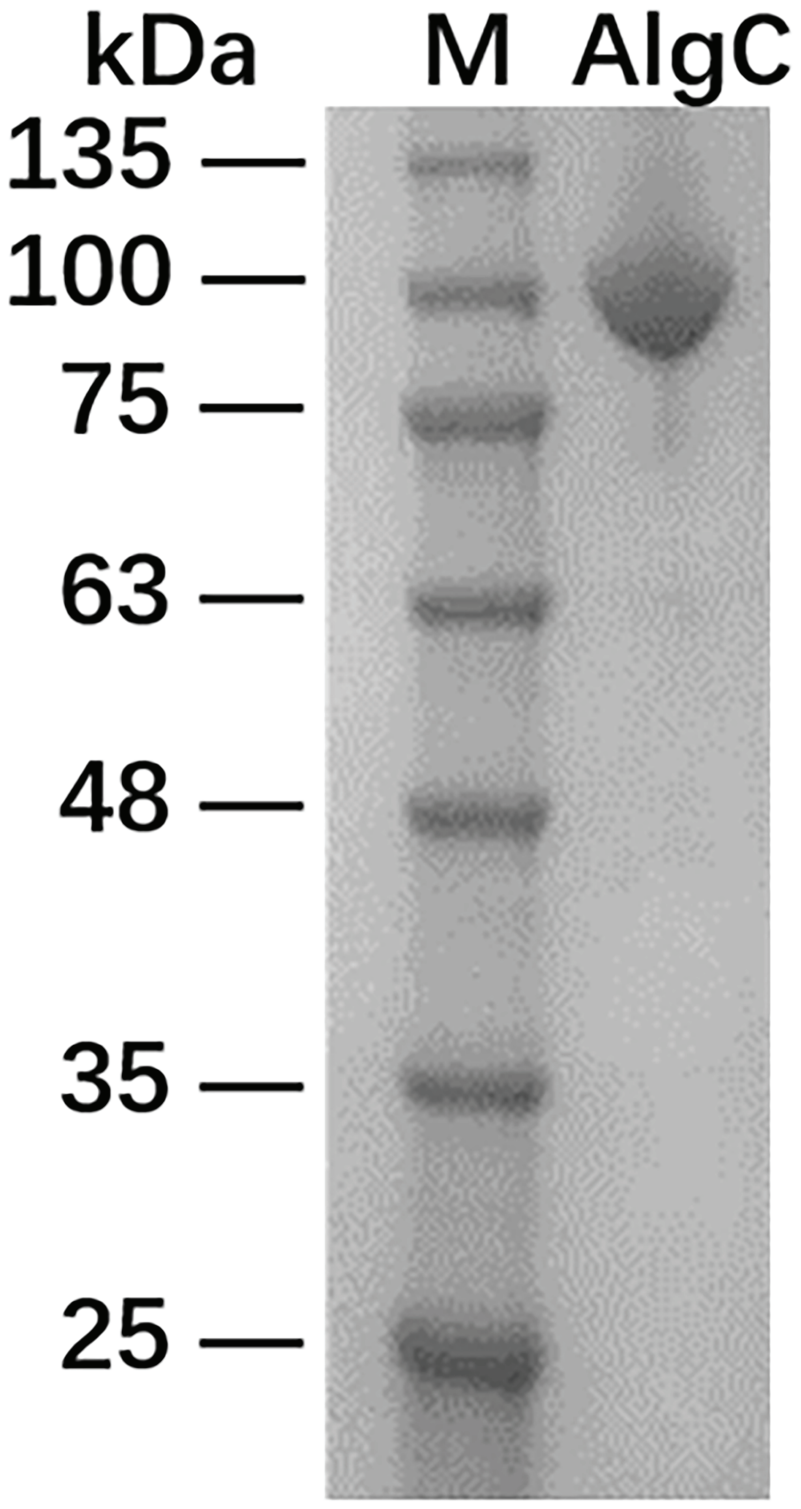
SDS-PAGE analysis of maltose-binding protein-10L6AlgC fusion protein. Lane 1: protein ladder; Lane 2: fusion protein.

The recombinant 10L6AlgC protein containing MBP at its N-terminus was induced with IPTG and 35 mg of purified fused protein was obtained from 500 ml of culture medium. The recombinant protein was subsequently separated following SDS-PAGE electrophoresis, and exhibited a single band at approximately 92.81 kDa (Figure 2) comprising a ~43 kDa MBP (Malhotra, 2009) and ~50 kDa predicted molecular weight of 10L6AlgC.^3^

### Enzyme assays and kinetic properties of 10L6AlgC

**Figure 3:**
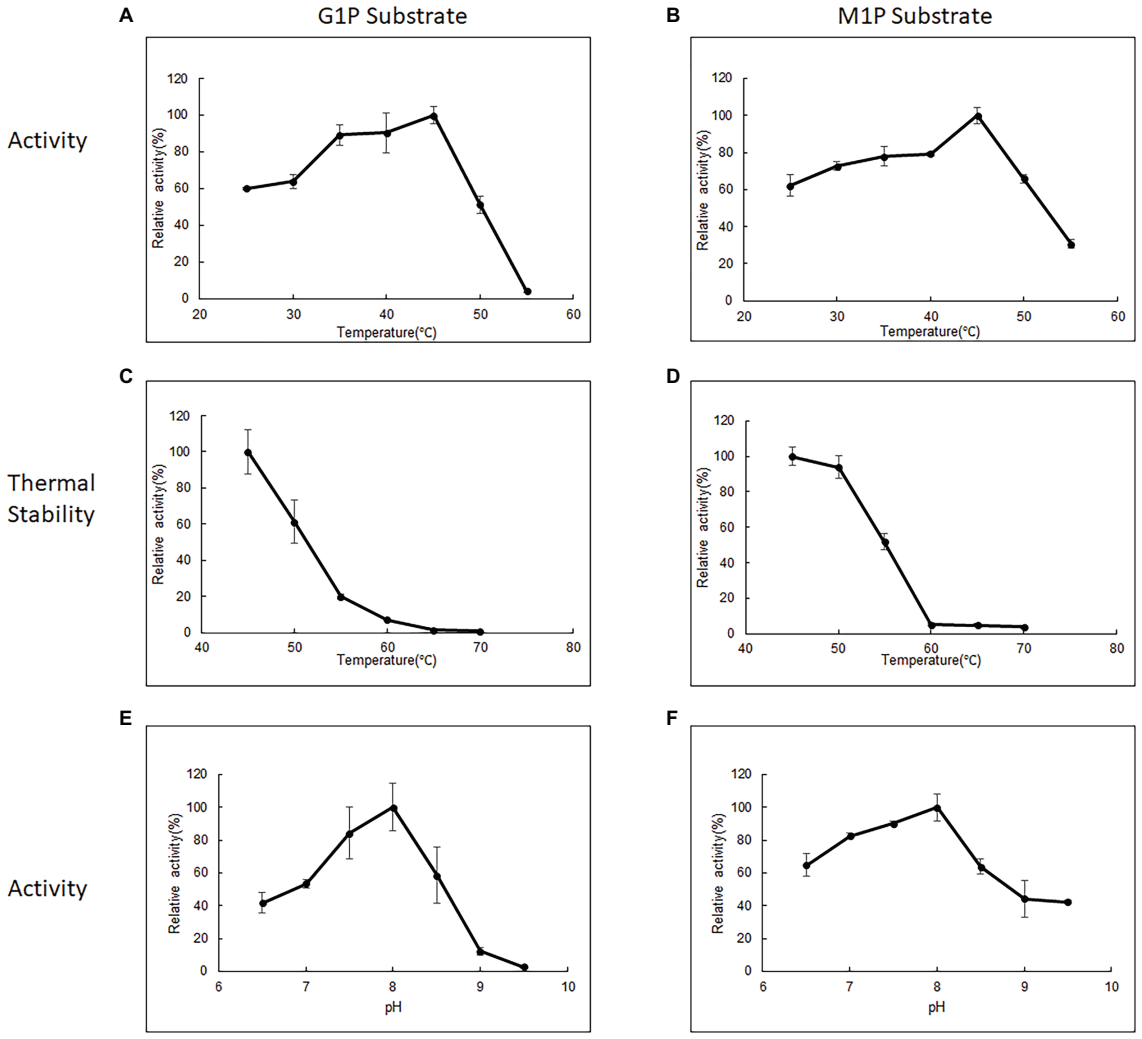
The influence of temperature **(A,B)**, thermal stability **(C,D),** and influence of pH **(E,F)** on 10L6AlgC activity. The relative **(A)** PGM activity and **(B)** PMM activity of AlgC at different temperatures (25–55°C). Relative thermal stability of **(C)** PGM and **(D)** PMM. Relative activity of **(E)** PGM and **(F)** PMM at different pH values (6.5–9.5). G1P substrate refers to PGM activity, M1P substrate refers to PMM activity.

Purified 10L6AlgC at a concentration of 2.1 mg/ml and of 0.106 mg/ml were used in the PGM and PGM enzyme assays, respectively. 10L6AlgC could optimally use both G1P (Figure 3A) and M1P (Figure 3B) as substrates at a temperature of 45°C, with a pH optimum of 8.0 being observed for both PGM and PMM activity (Figures 3E,F).

The 10L6AlgC enzyme was stable at 45°C, with a reduction in relative PGM activity of 61.36%. 19.7, 7.17, 1.43, and 0.72%, respectively, being observed for every 5°C increase in temperature up to 70°C (Figure 3C). In contrast, the PMM activity in 10L6AlgC was slightly less influenced by increases in temperature, with relative activities being reduced to 93.84, 51.66, 5.21, 4.98, and 3.79%, respectively; over the sample temperature range (Figure 3D).

**Figure 4:**
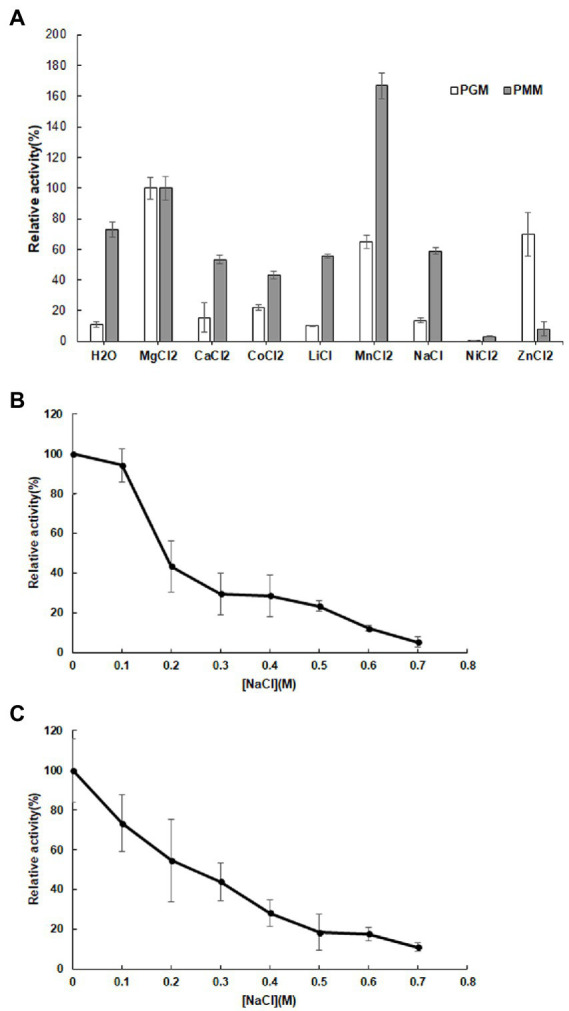
Influence of metal ions **(A)** on the relative activity of 10L6AlgC PGM and PMM activities; Influence of NaCl concentration on 10L6AlgC **(B)** PGM and **(C)** PMM relative activity.

Various metal ions displayed different effects on enzyme activity with Mg^2+^ promoting both PGM and PMM activity (Figure 4A). Mn^2+^ and Zn^2+^ also enhanced PGM activity, with Zn^2+^ in particular promoting PGM activity. Conversely, the presence of Ca^2+^, Co^2+^, Li^2+^, and Na^+^ had a negative effect on PMM activity relative to the control (Figure 4A).

**Figure 5:**
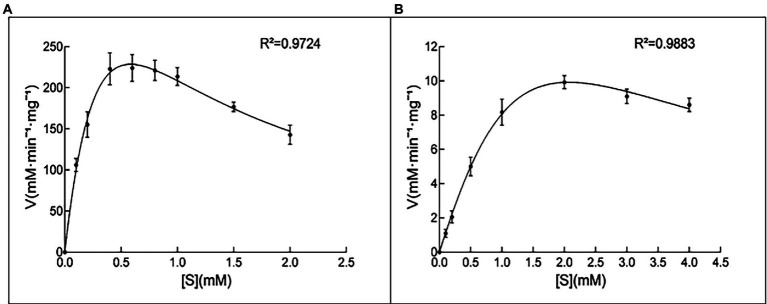
Kinetic analysis of 10L6AlgC. **(A)** G1P as substrate; **(B)** M1P as substrate. **(A)** The catalytic rates were measured in standard mixture with various concentrations of G1P (0, 0.1, 0.2, 0.4, 0.6, 0.8, 1, 1.5 and 2 mM) under optimal conditions; the measured *K*_m_ and *V*_max_ were 0.5314 and 644.7 mM min^−1^ mg^−1^. **(B)** Determination of the *K*_m_ for the substrate M1P. The catalytic rates were measured in standard mixture with various concentrations of M1P (0, 0.1, 0.2, 0.5, 1, 2, 3, and 4 mM) under optimal conditions; the measured *K*_m_ and *V*_max_ were 2.229 and 29.35 mM min^−1^ mg^−1^.

Both the 10L6AlgC PGM and PMM activities decreased at increased NaCl concentrations, with PMM displaying slightly higher levels of relative activity (55% relative activity at 0.2 M NaCl) than PGM (43% relative activity at 0.2 M); but as NaCl concentrations increased, similar decreases in relative PGM and PMM activities were evident (Figures 4B,C). Finally, the *K*_m_ and *V*_max_ values for 10L6AlgC were determined for each substrate, with 0.106 × 10^−2^ mg and 2.1 × 10^−2^ mg purified 10L6AlgC being used in the PGM and PMM reaction mixtures, respectively. A *K*_m_ of 0.5314 mM and a *V*_max_ of 644.7 mM min^−1^ mg^−1^ for G1P were observed (Figure 5A), while the *K*_m_ value and *V*_max_ of 10L6AlgC for M1P were determined as 2.229 mM and 29.35 mM min^−1^ mg^−1^ for M1P (Figure 5B). The *K*_cat_ of 10LAlgC was 99.72 S^−1^ for G1P and 4.45 S^−1^ for M1P, and the *K*_cat_/*K*_m_ were 187.66 and 2.04 mM^−1^ s^−1^, respectively.

### Protein modeling

**Figure 6:**
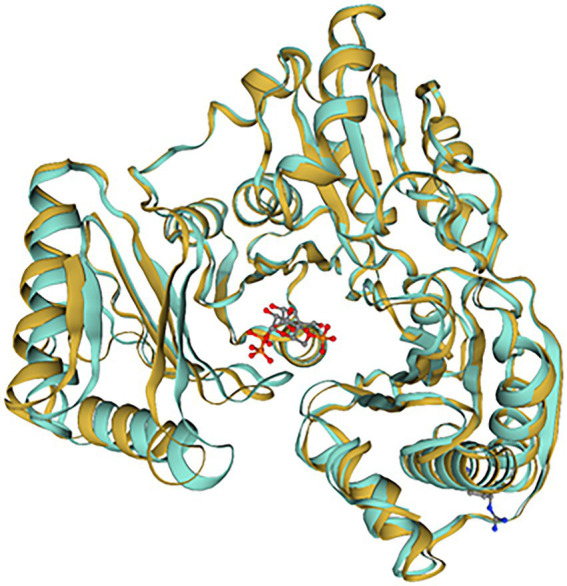
SWISS-MODEL comparison of 10L6AlgC and the closest related template (2h4l). 10L6AlgC is goldenrod-colored, 2h4l is turquoise-colored. The ligand, 1-O-phosphono-alpha-d-ribofuranose (R1P), is shown as ball-and-stick.

10L6AlgC was modeled using the SWISS-MODEL server, based on the top template, a 463 amino acid PMM/PGM enzyme, from *Pseudomonas aeruginosa* PAO1 (PDBe code: 2h4l) (Regni et al., 2006), with bound ligands (1) Zn and (2) 1-O-phosphono-alpha-d-ribofuranose (R1P) (Figure 6). The 10L6AlgC model displayed a GMQE score of 0.88 and a QMEAN *Z*-score of 0.53, indicating a high-quality, reliable model. The ERRAT Overall Quality Factor was calculated to be 94.7% and a VERIFY averaged 3D-1D score (≥0.2) of 100%, exceeding the threshold values in both cases. The overall PROCHECK *G*-score was predicted to be −0.12 (negative value desired) and the ProSA *Z*-score was −8.15 (within native conformation range), with both indicating good model quality. PROCHECK Ramachandran plot calculations revealed 92.6% of amino acid residues in the most favored regions, with an additional 7% in allowed regions, and 0% in disallowed regions.

## Discussion

In this study, we employed a metagenomics-based approach to explore the biocatalytic potential of the microbiome of the macroalga *L. digitata.* Previous reports had highlighted that bacteria associated with macroalgae such as *A. nodosum* are enriched in macroalgal-polysaccharide-degrading bacteria (Ihua et al., 2019), some of which produce algal cell-wall degrading enzymes to help mobilize polymers for nutritional purposes (Martin et al., 2015), particularly when growing in a nutrient-limited state such as during algal decay, and in doing so contribute to algal biomass recycling (Chun et al., 2017). Thus we deduced that if *L. digitata* was allowed to decay under controlled conditions at different temperatures that this would be likely to result in an enrichment of bacteria from its microbiota that produced algal cell wall polysaccharidases, as a result of the nutrient-limited state to which the microbial populations had been exposed. We had previously used a similar approach with decaying *A. nodosum* which had resulted in such an enrichment (Ihua et al., 2019). Following screening and subsequent sequencing of a clone from the metagenomic library, a gene encoding a bifunctional PMM/PGM was identified. PGM and PMM are members of the alpha-d-phosphohexomutase superfamily of enzymes and are ubiquitous in the three kingdoms of life. They catalyze the reversible conversion of 1-phospho to 6-phosphohexoses, involving various different sugars such as glucosamine, glucose, mannose, and N-acetylglucosamine (Stiers et al., 2017). Bacterial members of the family are typically involved in the biosynthesis of a variety of carbohydrates such as exopolysaccharides, lipopolysaccharides (LPSs), and alginate (Stiers et al., 2019).

PGM (E.C. 5.4.2.2) is involved in the reversible interconversion of glucose-1-phosphate (G1P) and glucose-6-phosphate (G6P) and is a key enzyme linking the glycolytic and gluconeogenesis pathways in bacteria (Wang and Zhang, 2010). When involved in the synthesis of G1P, UDP-glucose is subsequently formed which is an important precursor for many biosynthetic pathways. PMM reversibly catalyzes the interconversion of mannose-6-phosphate to M1P, which is a key metabolic precursor for the production of GDP-d-Mannose, that is used in the production of structural carbohydrates, LPSs, and exopolysaccharides (Coyne et al., 1994; Levander and Radstrom, 2001; Yang et al., 2010) in many bacteria and in the alginate biosynthetic pathway in *Pseudomonas aeruginosa* (Coyne et al., 1994; Ye et al., 1994; Hay et al., 2013). GDP-d-mannose is also a key metabolic intermediate in the production of various glyco-conjugates such as GDP-l-fucose, GDP-d-rhamnose in *P. aeruginosa* (Olvera et al., 1999; Maki et al., 2002), and GDP-d-talose in *Actinobacillus actinomycetemcomitans* (Suzuki et al., 2002).

LPS and alginate biosynthetic pathways, involving *algC*, are key virulence factors in *P. aeruginosa* (Pedersen et al., 1992; Pier, 2007), in *Mycobacterium tuberculosis* (*M. tb*) (McCarthy et al., 2005) and in *Helicobacter pylori* (Liu et al., 2022). *P. aeruginosa* PAO1 mutants with a disrupted PMM displayed significantly reduced virulence in mouse models (Tang et al., 1996). *M. tb* interaction with macrophages is mediated by bacterial mannose containing glycoconjugates and immune cell mannose receptors. McCarthy and colleagues reported that overexpression of PMM resulted in greater interaction between *Mycobacterium smegmatis* and human macrophages (McCarthy et al., 2005). PMM/PGM also plays a role in the biosynthesis of fucose in *H. pylori* and is a key constituent of the O-antigen in that pathogen. PMM/PGM knockouts in *H. pylori* (together with knockouts of GDP-d-mannose dehydratase) resulted in almost complete loss of the O-antigen polysaccharide (Liu et al., 2022). Thus, targeted and specific disruption of bacterial PMMs may be an attractive drug target worthy of investigation. Using this hypothesis, Zhu and co-workers synthesized structural analogs of the enzyme’s substrates and identified 4 structures that were effective competitive inhibitors of PMM/PGM (Zhu et al., 2019). PGM activity has been reported to be involved in capsular polysaccharide (CPS) biosynthesis in the opportunistic bacterial pathogen *Streptococcus pneumoniae*, where it is involved in the reversible isomerization of G6P to G1P, with G1P being required to generate UDP-glucose, a sugar precursor for CPS (Paton and Trappetti, 2019). The production of CPS is a key virulence determinant for the survival and pathogenesis of *S*. *pneumoniae* in the host (McFarland et al., 2021). α-PGM has been reported to play an important role in carbohydrate metabolism and to be essential for the growth of *Lactococcus lactis* (Neves et al., 2006). PMM gene expression in the marine bacterium *Pseudoalteromonas agarivorans* has recently been shown to be upregulated in response to changes in pH, resulting in increased exopolysaccharide production in the strain (Ju et al., 2022). This increase in PMM expression is accompanied by a decrease in PGM expression levels, thereby increasing the conversion of glucose-6-P to mannose-1-P, ultimately leading to increased GDP-l-fucose production in the strain (Ju et al., 2022). EPS from *P. agarivorans* have previously been reported to exhibit good antioxidant activity (Hao et al., 2018).

PGM has recently been shown to play a major role in xyloglucan catabolism in the anaerobic Gram-positive bacterium *Ruminiclostridium cellulolyticum*, where it converts a-d-glucose-1-phosphate (Glu1P) produced by both cellobiose phosphorylase and by the Leloir pathway, into d-glucose 6-phosphate (Glu6P), which then enters the glycolytic pathway (Kampik et al., 2021).

Apart from their involvement in carbohydrate metabolism in bacteria, PMM/PGMs have also been shown to have other quite diverse roles with an AlgC-type PMM activity being shown to be involved in the production of type IV pili and linked to twitching mobility in *Lysobacter enzymogenes* (Qian et al., 2019); while PGM activity in the citrus canker causing bacterium *Xanthomonas citri* is required for the synthesis of precursors of the pathogenesis-related polysaccharide xanthan (Goto et al., 2016). PGM activity has also been shown to be involved in normal cell growth and morphology, together with biofilm formation in *B. subtilis* (Lazarevic et al., 2005). While PMM expression has been linked to production of the antibiotic bleomycin, with regulation of the expression of N-acetylglucosamine in *Streptomyces verticillus* by genetically engineering the GDP-mannose biosynthetic pathway, through increased expression of PMM isomerase (*manA*) and PMM (*manB*) genes in the strain resulting in enhanced bleomycin production in the strain (Chen et al., 2020).

In addition PGM has been reported to be involved in hyaluronic acid (HA) biosynthesis in the animal pathogen *Pasteurella multocida*. These bacteria produce capsules which contain a number of different types of polysaccharides, depending on their capsular serotypes, with serotype A containing HA. In *P. multocida* HA biosynthesis involves the conversion of glucose-6-phosphate to glucose-1-phosphate by PGM; which is ultimately then converted to UDP-glucuronic acid, an important HA precursor in the strain (Pasomboon et al., 2021).

### Phylogeny of 10L6AlgC

The gene sequence encoding the 10L6AlgC protein is identical to that in the sequenced genome of *Cobetia* sp. L2A1 and given the high similarity between the ~35 Kb cloned fosmid insert and the *Cobetia* sp. L2A1 genome sequence (99% identity), it is probable that the cloned DNA is from the same species. *Cobetia* sp. L2A1 was originally isolated from a brown algae and first identified in an alginate utilization screen (Cha et al., 2021), and is a member of the family Halomonadaceae which can form biofilms which involves aggregation and the production of exopolysaccharides (Shea et al., 1991) that can influence the secondary colonization of invertebrates and algae (Shea et al., 1995). Phylogenetic analysis of the 10L6AlgC shows that the protein is conserved within the genus (96–100% amino acid sequence identity) as revealed by BLAST analysis. The most similar proteins outside of *Cobetia* spp., are clades of PMM/PGMs from *Chromohalobacter* spp. and *Halomonas* spp. (78–80% amino acid sequence identity) from the same family (Halomonadaceae, phylum Pseudomonadota) (Figure 1). Structural alignments (data not shown) reveal that the ligand (substrate and metal ion) binding sites and catalytic sites are conserved between these 3 genera and that the protein differences are on the surface of the proteins. While differences between the amino acid sequences of the three aforementioned genera are apparent, the sequences are conserved within each individual genus and may be considered characteristic of the genus. Interestingly, these are all halophilic or moderately halophilic species. While increasing concentrations of NaCl resulted in decreased PGM and PMM activities in 10L6AlgC, the enzyme was predicted to be localized to the cytoplasm; so ambient marine salinity is unlikely to affect enzyme activities.

### Biochemical characterization

Several PGM/PMM mono- and bi-functional enzymes have to date been biochemically characterized, including bacterial enzymes from *E. coli* (Joshi and Handler, 1964), *P. aeruginosa* (Ye et al., 1994), *Acetobacter xylinum* (Kvam et al., 1997), and *Clostridium thermocellum* (Wang and Zhang, 2010), together with archaeal enzymes from the hyperthermophiles *Thermococcus kodakaraensis* (Rashid et al., 2004) and *Pyrococcus horikoshii* (Akutsu et al., 2015). The protein from our study has a similar molecular weight to other PGM/PMM enzymes that have been characterized to date, which typically range from 48–65 kDa. The predicted molecular weight of 10L6AlgC is ~50 kDa and is thus very similar to those from *P. aeruginosa* (50 kDa) (Ye et al., 1994), *T. kodakaraensis* (48–50 kDa) (Rashid et al., 2004), and *P. horikoshii* (50 kDa). It is however smaller than those from *E. coli* (62 kDa) (Joshi and Handler, 1964), *A. xylinum* (59 kDa) (Kvam et al., 1997), and *C. thermocellum* (65 kDa) (Wang and Zhang, 2010). The *E. coli* and *A. xylinum* enzymes are mono-specific PGMs, while the *P. aeruginosa*, *C. thermocellum*, *T. kodakaraensis*, and *P. horikoshii* are bi-functional enzymes, which like 10L6AlgC display both PMM and PGM activities.

The *K*_m_ of the 10L6AlgC PGM activity (0.5314 mM) was greater than that of many of the aforementioned enzymes; such as *E.coli* (0.05 mM) (Joshi and Handler, 1964), *P. aeruginosa* (0.022 mM) (Ye et al., 1994), *A. xylinum* (0.0026 mM) (Kvam et al., 1997), *C. thermocellum* (0.41 mM) (Wang and Zhang, 2010), as well the archaeal enzyme from *P. horikoshii* (0.09 mM) (Akutsu et al., 2015). It was however lower than the other biochemically characterized archael PGM from that of the *T. kodakaraensis* (3.0 mM) (Rashid et al., 2004). Similarly, the *K*_m_ for PMM activity in 10L6AlgC was greater than that of (*P. aeruginosa* [0.017 mM], *C. thermocellum* [0.44 mM], and *P. horikoshii* [0.09 mM]), but again as with the PGM activity was lower than that of *T. kodakaraensis* (3.2 mM).

With respect to optimal pH, the optimum pH for 10L6AlgC activities was 8.0, which was slightly less alkaline than the aforementioned enzymes which ranged from pH 8.8–9.0; such as those from *E. coli* (pH 9.0) (Joshi and Handler, 1964), *C. thermocellum* (pH 8.8) (Wang and Zhang, 2010), and *P. horikoshii* (pH 9.0) (Akutsu et al., 2015). In contrast, the optimal pH was more alkaline than those from *A. xylinum* (pH 5.5–7.4) (Kvam et al., 1997) and *T. kodakaraensis* (pH 7.0).

Divalent cations are essential co-factors for PMM/PGM activity in 10L6AlgC. Relative to activity in the presence of Mg^2+^, Mn^2+^ increased the PMM activity but decreased the PGM activity. Reduced enzyme activities were also seen in the presence of Ca^2+^, Co^2+^, Li^2+^, Na^2+^, Ni^2+^, and Zn^2+^. This PMM/PGM activity profile differs from the activity profile from the aforementioned characterized enzymes in other strains. For example in *E. coli* Ni^2+^ and Zn^2+^ did not affect PGM activity, while Mn^2+^ resulted in reduced activity (Joshi and Handler, 1964). In the very well-characterized PMM/PGM enzyme from *P. aeruginosa* for which a crystal structure is available, a bound Mg^2+^ ion is known to be required for maximal activity and is believed to function in activating the transfer of a phosphoryl group (Rashid et al., 2004). In *P. aeruginosa,* Mn^2+^ and Ca^2+^ greatly reduced PGM and PMM activities, while Co^2+^, Ni^2+^, Zn^2+^, and Li^2+^ completely abolished activities. Similarly, the addition of Ca^2+^, Co^2+^ and Ni^2+^ completely abolished enzyme activities in *P. horikoshii* (Akutsu et al., 2015), while increased activities were observed in *T. kodakaraensis* in the presence of Mn^2+^, Ni^2+^, and Zn^2+^ (Rashid et al., 2004). Similar to PGM activities from *A. xylinum* the addition of anions in the form of Cl^−^ (NaCl in this study, HCL in Kvam et al., 1997), decreased 10L6AlgC activities at increasing concentration (Figure 4B). The optimum temperature for PGM and PMM activities in 10L6AlgC (45°C) is understandably lower than those from the archaeal thermophiles and for the PMM/PGM enzyme from the thermophile *C. thermocellum* (70°C) (Wang and Zhang, 2010); but is higher than the optimum temperature for the *P. aeruginosa* enzyme (<37°C) (Ye et al., 1994).

### Protein modeling

**Figure 7:**
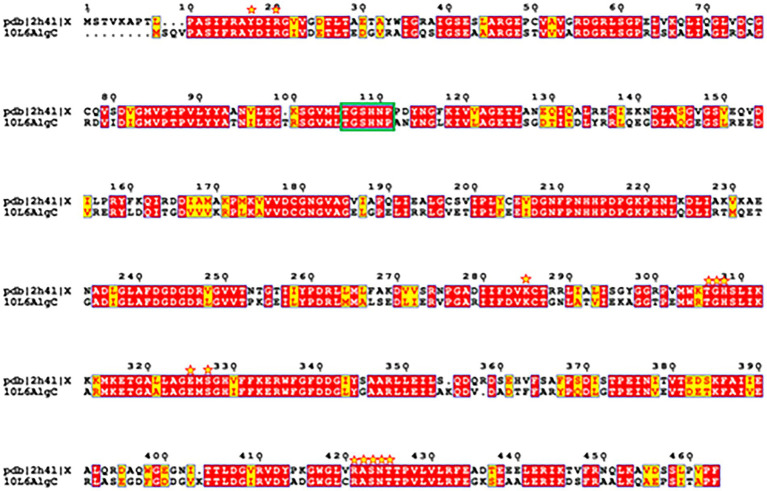
Amino acid sequence structural alignment of 10L6AlgC and 2h4l generated with T-COFFEE Expresso and rendered using ESPript 3.0. Amino acid residues shaded in red represent the ones strictly conserved while residues highlighted in yellow depict areas with an average level of homology. Residues which interact with the carbohydrate ligand are marked with yellow stars. Catalytic residues are marked with a green box.

**Figure 8:**
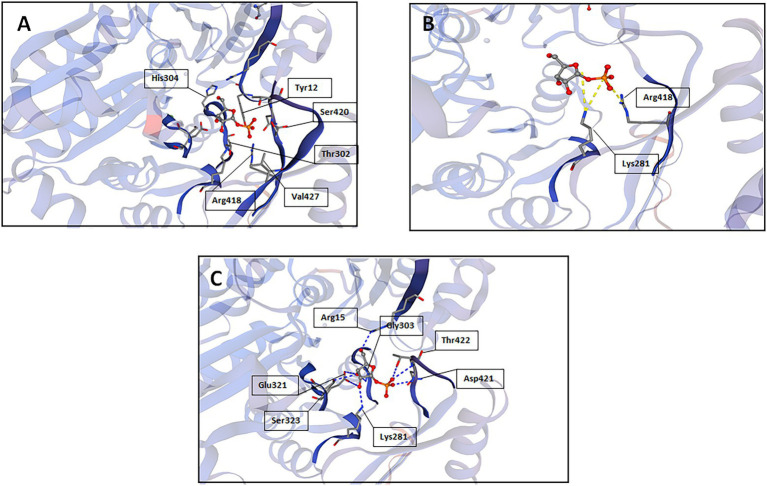
SWISS-MODEL predictions of 10L6AlgC amino acid residues which interact with the carbohydrate substrate; **(A)** interactions other than hydrogen bonds or salt bridges, **(B)** salt bridges, and **(C)** hydrogen bonds.

A reliable model of the enzyme was constructed by SWISS-MODEL and revealed that hydrogen bonding, ligand-binding and catalytic residues were conserved between the model and the template (Figure 6) despite only an overall 62% amino acid sequence identity. Further inspection showed ~36% of the amino acid substitutions possessed homologous biochemical properties (hydrophobicity, hydrophilicity, charge) (Figure 7). The overall tertiary structure of 10L6AlgC is highly similar to the template structure (Figure 6). Thirteen residues interact with the carbohydrate ligand (Tyr12, Arg15, Lys281, Thr302, Gly303, His304, Glu321, Ser323, Arg418, Ser420, Asn421, Thr422, and Val427) (Figure 8) and these sites are conserved between the model and the template (Figure 7). Structural alignments of the amino acid sequences of the model and the template (Figure 7) and also of 10L6AlgC and closely related proteins from *Cobetia* spp., *Chromohalobacter* spp., and *Halomonas* spp. (data not shown) reveal that the metal co-factor binding (DGDGD) and catalytic site (TGSHNP) motifs are strictly conserved in the family. Of the 13 amino acids interacting with the substrate and ligand, 7 of them form 10 H-bonds (Figure 8C) (Arg15, Lys281 [2 x H-bonds], Gly303, Glu321 [2 x H-bonds], Ser323, His421, and Thr422 [2 x H-bonds]). Lys281 plays an important role in substrate docking as the two hydrogen bonds also form salt bridges with the hexose sugar and the phosphate when attached to C1 (Figure 8B), as predicted by the model.

### Biotechnological potential

PMM/PGMs have received interest over the years in the context of their use in enzyme-based production of biochemicals with different potential industrial applications (Wei et al., 2021; Dai et al., 2022). For example, a heat stable PGM which has been characterized in *Clostridium thermocellum* (Wang and Zhang, 2010) has recently been used to convert glucose 1 phosphate to glucose 6-phosphate in an enzymatic cascade involving five other enzymes in a one-pot stoichiometric conversion of starch to mannitol (Wei et al., 2021). The production of glucose 6-phosphate by PGM in this way is a key step in the recently established *in vitro* system for the production of mannitol, a low-calorie sweetener in the food industry, from low-cost starch-based materials. This system has the potential to be further expanded to use starch for the efficient synthesis of other value-added biochemicals (Wei et al., 2021). Similarly, the PGM from *Thermococcus kodakaraensis* has also recently been used in a recombinant *E. coli* vector system for the production of another food sweetener, namely *D*-tagatose from maltodextrin using whole-cell catalysts (Dai et al., 2022); while a thermostable PGM from *T. kodakaraensis* has been used in the production of myo-inositol, an important human dietary supplement, from starch (Fujisawa et al., 2017). A recombinant PGM from *E. coli* has also been used to enhance UDP-galactose derived synthesis of the bioactive compound hyperoside (quercetin 3-*O*-galactoside) in a metabolically engineered *E. coli* strain (Li et al., 2022). In addition, PGM from *T. kodakarensis* has recently been heterologously expressed in *B. subtilis* thereby allowing for the recombinant enzyme to be produced at high levels in high-density fermentations for subsequent potential synthesis of inositol from starch (Ye et al., 2022). Increased expression of PGM to generate increased polysaccharide production has recently been achieved in the fungus *Cordyceps militaris*, with homologous overexpression of PGM and UDP-glucose 6-dehydrogenase in the fungus resulting in an approximately 78% increase in polysaccharide levels. These fungal polysaccharides are known to have protective effects on both the kidney and liver (Wang et al., 2021).

The biochemical characteristics of 10L6AlgC may have useful industrial biotechnological applications. In particular, the temperature (45°C) and pH (8.0) optima may be of greater interest than currently utilized enzymes (*C. thermocellum* [70°C; pH 8.8] (Wang and Zhang, 2010); *T. kodakaraensis* [90°C, pH 7.0] (Rashid et al., 2004); engineered *E. coli* [20°C] (Li et al., 2022)). Thus, it is clear that 10L6AlgC possesses a unique biochemical profile when compared to previously characterized PMM/PGM bifunctional enzymes and may find utility in enzyme-based production of biochemicals with different potential industrial applications, in which other bacterial PMM/PGMs have previously been used.

## Data availability statement

The raw fosmid sequence read data can be found in the GenBank Short Read Archive (SRA) with the Accession No. SAMN29845194. The *10L6algC* gene nucleotide and protein amino acid sequences can be found in GenBank with the Accession No. ON876002.

## Author contributions

SJ, MI, MD, and PZ performed the experimental work. SJ, MD, PZ, and DD analyzed the data. All authors conceived and designed the experiments and contributed reagents, materials, and analysis tools. SJ, DD, and AD wrote the manuscript. DS, DD, and AD supervised the study. All authors contributed to the article and approved the submitted version.

## Funding

This work was supported by the Qingdao International Innovation Cooperation Project for Science and Technology (no. 22-3-6-ghgg-1-hz), the National Nature Science Foundation of China (no. 42006101), the Marine Biotechnology ERA/NET, NEPTUNA project (contract no. PBA/MB/15/02), the Irish Department of Agriculture, Food and the Marine (DAFM) SMI-BIO project (15/F/698), and the BlueBioCOFUND project MINERVA.

## Conflict of interest

The authors declare that the research was conducted in the absence of any commercial or financial relationships that could be construed as a potential conflict of interest.

## Publisher’s note

All claims expressed in this article are solely those of the authors and do not necessarily represent those of their affiliated organizations, or those of the publisher, the editors and the reviewers. Any product that may be evaluated in this article, or claim that may be made by its manufacturer, is not guaranteed or endorsed by the publisher.

## Supplementary material

The Supplementary material for this article can be found online at: https://www.frontiersin.org/articles/10.3389/fmicb.2022.1000634/full#supplementary-material

## Footnotes

^1^ https://www.graphpad.com/scientific-software/prism/

^2^ http://wishart.biology.ualberta.ca/basys

^3^ https://www.aatbio.com/tools/calculate-peptide-and-protein-molecular-weight-mw
