# Supplementary material for: Isolation, identification, and biochemical characterization of a novel bifunctional phosphomannomutase/phosphoglucomutase from the metagenome of the brown alga Laminaria digitata
Source: Front Microbiol. 2022 Sep 23;13:1000634. doi: 10.3389/fmicb.2022.1000634 (PMC9537760; doi:10.3389/fmicb.2022.1000634)
Supplement: Supplementary file 1 [file Table_1.DOCX]

| **Start** | **End** | **Strand** | **Gene** | **COG** | **Protein Function** |
| --- | --- | --- | --- | --- | --- |
| 5896 | 3107 | **-** | copA | COG2217 | Copper-exporting P-type ATPase A |
| 6348 | 5893 | **-** | zntR | COG0789 | HTH-type transcriptional regulator zntR homolog |
| 6783 | 8048 | **+** | fadL | COG2067 | Long-chain fatty acid transport protein |
| 9585 | 8230 | **-** | yedS | COG3203 | Outer Membrane Porin |
| 11428 | 11547 | **+** | - | - | Hypothetical Protein |
| 11438 | 9675 | **-** | yejF | COG4172 | Uncharacterized ABC transporter ATP-binding protein YejF |
| 12375 | 11452 | **-** | oppC | COG1173 | Oligopeptide transport system permease protein oppC |
| 13314 | 12388 | **-** | oppB | COG0601 | Oligopeptide transport system permease protein oppB |
| 15262 | 13436 | **-** | mppA | COG4166 | Periplasmic murein peptide-binding protein |
| 16958 | 15279 | **-** | mppA | COG4166 | Periplasmic murein peptide-binding protein |
| 17568 | 18449 | **+** | prfB | COG1186 | Peptide chain release factor 2 |
| 18618 | 20156 | **+** | lysS | COG1190 | Lysyl-tRNA synthetase |
| 20422 | 20853 | **+** | - | - | Conserved Hypothetical Protein |
| 21024 | 21371 | **+** | bolA | COG0271 | Protein BolA |
| 21374 | 22354 | **+** | - | - | Hypothetical Protein Csal |
| 22351 | 22845 | **+** | rimI | COG0456 | Ribosomal-protein-alanine acetyltransferase |
| 23253 | 22978 | **-** | zapB | COG3074 | Cell division protein ZapB |
| 24136 | 23462 | **-** | rsmD | COG0742 | Ribosomal RNA small subunit methyltransferase D |
| 24153 | 25970 | **+** | ftsY | COG0552 | Cell division protein ftsY |
| 25967 | 26635 | **+** | ftsE | COG2884 | Cell division ATP-binding protein FtsE |
| 26632 | 27666 | **+** | ftsX | COG2177 | Cell division protein ftsX homolog |
| 27913 | 28788 | **+** | rpoH | COG0568 | RNA polymerase sigma-32 factor |
| 28964 | 31594 | **+** | yciR | COG2200 | Uncharacterized signaling protein PA1727 |
| 32361 | 31744 | **-** | slmA | COG1309 | HTH-type protein slmA |
| 33489 | 32476 | **-** | argB | COG0548 | Acetylglutamate kinase |
| **34843** | **33572** | **-** | **algC** | **COG1109** | **Phosphomannomutase/phosphoglucomutase** |

Supplementary Table S1: Contig annotation using BASys web server.
