# Supplementary material for: Isolation, identification, and biochemical characterization of a novel bifunctional phosphomannomutase/phosphoglucomutase from the metagenome of the brown alga Laminaria digitata
Source: Front Microbiol. 2022 Sep 23;13:1000634. doi: 10.3389/fmicb.2022.1000634 (PMC9537760; doi:10.3389/fmicb.2022.1000634)
Supplement: Supplementary file 2 [file Data_Sheet_1.docx]

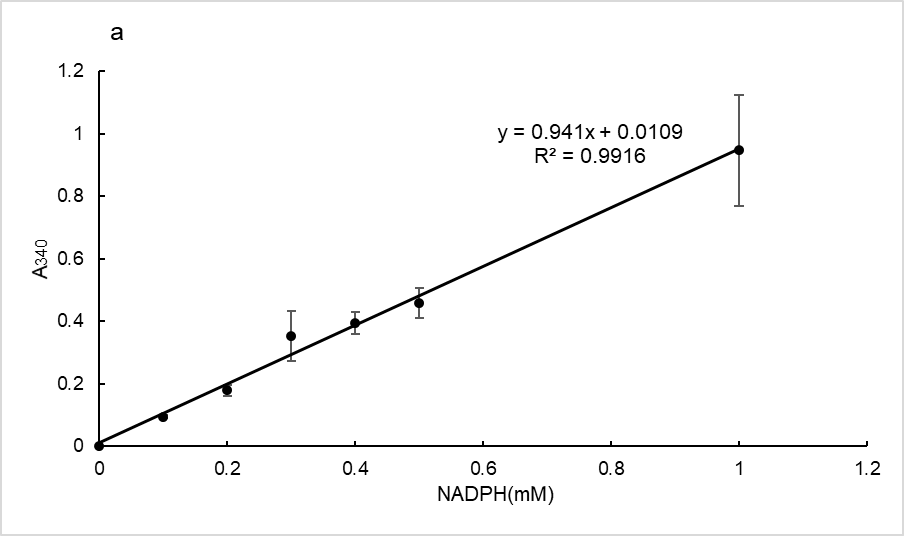


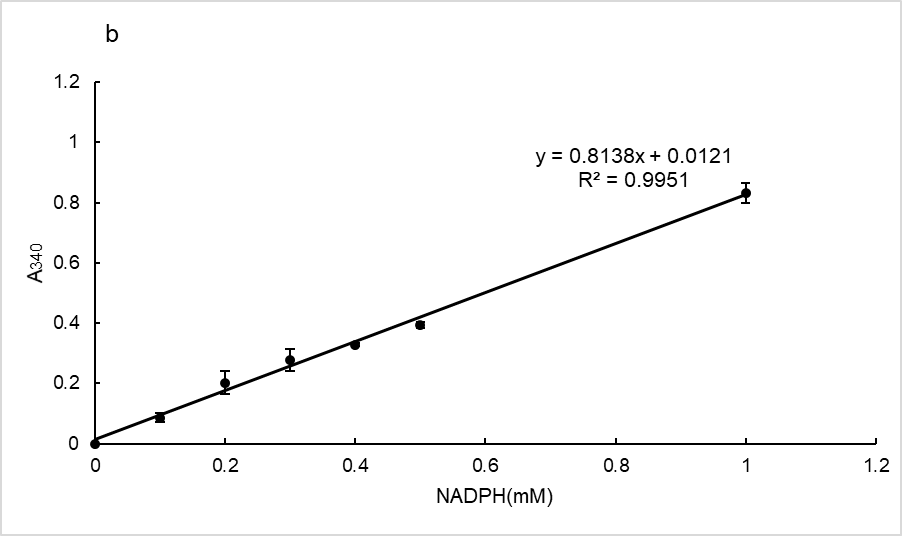


**Supplementary Figure S1**: Standard curve of NADPH for phosphoglucomutase/phosphomannomutase (PGM/PMM). (a) The standard curve for PGM; (b) The standard curve for PMM activity.
