# Supplementary material for: Isolation, identification, and biochemical characterization of a novel bifunctional phosphomannomutase/phosphoglucomutase from the metagenome of the brown alga Laminaria digitata
Source: Front Microbiol. 2022 Sep 23;13:1000634. doi: 10.3389/fmicb.2022.1000634 (PMC9537760; doi:10.3389/fmicb.2022.1000634)
Supplement: Supplementary file 3 [file Data_Sheet_2.docx]

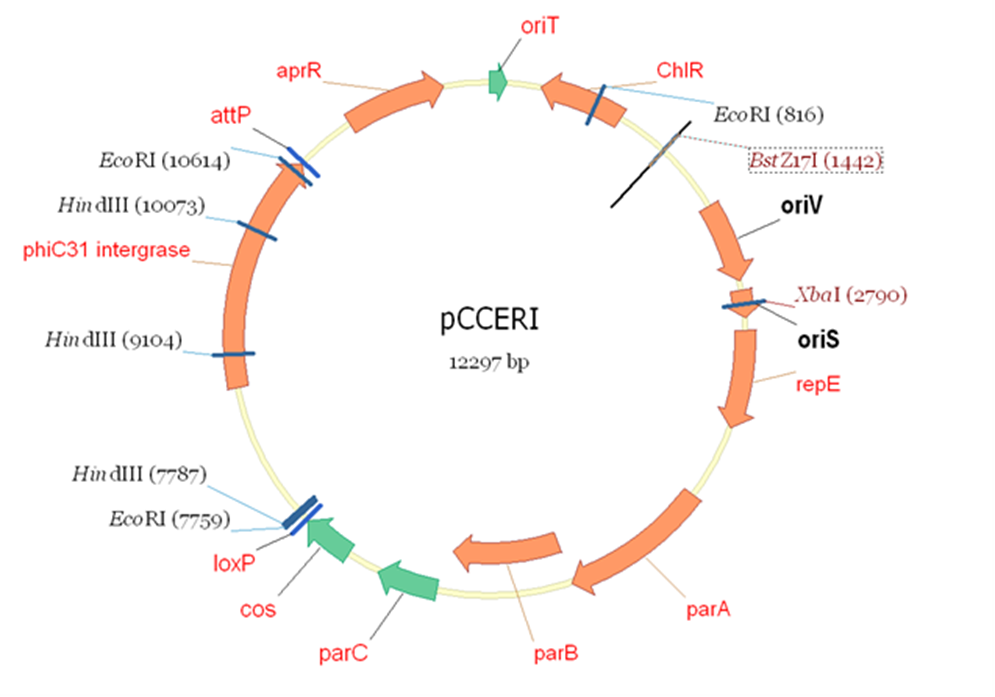


Supplementary Figure S2: pCCERI Fosmid map of cloning vector pCCERI derived from the commercial fosmid pCC1FOS.
