# Supplementary material for: Isolation, identification, and biochemical characterization of a novel bifunctional phosphomannomutase/phosphoglucomutase from the metagenome of the brown alga Laminaria digitata
Source: Front Microbiol. 2022 Sep 23;13:1000634. doi: 10.3389/fmicb.2022.1000634 (PMC9537760; doi:10.3389/fmicb.2022.1000634)
Supplement: Supplementary file 4 [file Data_Sheet_3.docx]

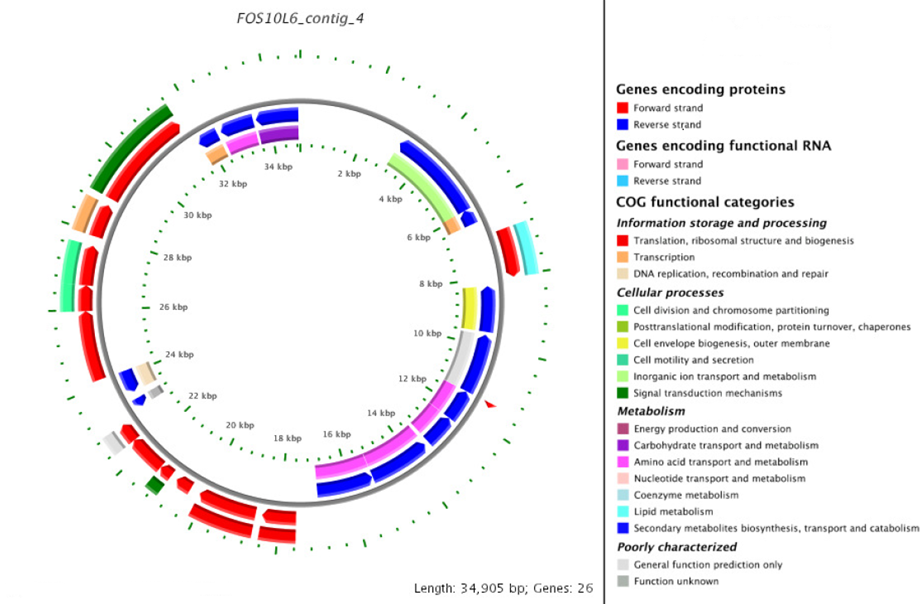


**Supplementary Figure S3**: circularised graphical representation of the contig 4 of the sequenced fosmid insert from clone 10L6. Protein encoding genes on the forward strand appear in blue inside the grey line; genes encoding proteins on the reverse strand appear in red outside the grey line. Genes are further illustrated as colour-coded by COG functional categories.
